# ORAL PLENARY SESSION 2: (Fellows Plenary)

**DOI:** 10.1002/pmf2.12009

**Published:** 2025-01-05

Abstracts 39 – 46

FRIDAY

January 31, 2025

8:00 AM – 10:00 AM

Aurora Ballroom

MODERATORS

Bryann Bromley, MD

Mark A. Clapp, MD, MPH

